# Oral Health-Related Factors Associated with Dysphagia Risk among Older, Healthy, Community-Dwelling Korean Adults: A Pilot Study

**DOI:** 10.3390/healthcare12020267

**Published:** 2024-01-20

**Authors:** Da-Som Lee, Hee-Eun Kim, Jun-Seon Choi

**Affiliations:** 1 Department of Dental Hygiene, Graduate School, Gachon University, Incheon 21936, Republic of Korea; judylove92@naver.com; 2 Department of Dental Hygiene, College of Medical Science, Gachon University, Incheon 21936, Republic of Korea; hekim@gachon.ac.kr

**Keywords:** buccinator muscle strength, dysphagia, hyposalivation, masticatory discomfort, oral health

## Abstract

Most previous studies addressing dysphagia examined individuals who already had diseases causing dysphagia and did not pay much attention to oral health conditions as a risk factor. This pilot study investigated 62 healthy adults aged 65 years or older who were living independently in the community, performed basic activities of daily living independently, and had no history of a causative disease of dysphagia to identify the factors associated with dysphagia risk, especially oral health. The Dysphagia Risk Assessment Scale was used to screen the patients for dysphagia. Hyposalivation was diagnosed by evaluating the unstimulated salivary flow rate, and orofacial muscle strength (anterior tongue elevation, buccinator muscle, and lip strength) was quantitatively measured using the Iowa Oral Performance Instrument. To analyze the factors associated with dysphagia risk, the Mann–Whitney test, Kruskal–Wallis test, and multiple logistic regression analyses were conducted. In the final regression model adjusted for sociodemographic characteristics, the oral health-related factors independently associated with dysphagia risk were buccinator muscle strength, hyposalivation, and subjective masticatory discomfort (*p* < 0.05). Therefore, our findings suggest that weak buccinator muscle strength, hyposalivation, and subjective masticatory discomfort are valuable indicators for the early detection of dysphagia in older, healthy, independent, community-dwelling adults.

## 1. Introduction

Swallowing is a complex process in which a food bolus formed by mastication and saliva secretion is transported from the mouth to the stomach [1]. Swallowing is crucial for water and nutrient intake and is essential for maintaining life because it protects the airways [2]. Anatomically, swallowing can be divided into three phases based on the location of the food bolus: oral, pharyngeal, and esophageal [2,3]. These phases should occur in sequence, and any problem during these phases is referred to as dysphagia [3]. Dysphagia, or swallowing difficulties, is defined as difficulties in swallowing food, liquid, or both due to disorders of the muscles or nerves that are involved in deglutition or due to anatomical or psychological factors [4,5]. If left untreated for long periods in older adults, dysphagia can cause serious complications such as malnutrition, dehydration, weight loss, aspiration pneumonia, and suffocation [6,7]. Furthermore, coughing, throat clearing, or choking during or immediately after eating, which are typical symptoms of dysphagia, fear of eating and drinking, and prolonged mealtime duration, can lead to a decrease in quality of life [7,8]. Therefore, early detection of dysphagia risk can greatly contribute to minimizing the chances of developing various physical or psychological complications. 

In general, dysphagia is classified into two types: oropharyngeal and esophageal [9]. Cerebrovascular or neurologic diseases such as stroke and Parkinson’s disease are the major conditions leading to oropharyngeal dysphagia, the type mainly observed in older adults [10]. Esophageal dysphagia is caused by motor disorders of the esophagus or structural disorders, such as esophageal stricture [11]. Furthermore, the literature has shown that tooth loss or oral dryness has a significant impact on the deterioration of swallowing function in individuals with dysphagia-inducing disease [12,13,14]. Some studies have reported on the prevalence of dysphagia according to the research participants and screening methods. A systematic review and meta-analysis suggested that while the global prevalence of dysphagia was 43.8% and increased with age, the rate was approximately 80% in patients with dementia, Alzheimer’s disease, or stroke [15]. According to several studies that examined older community-dwelling adults, the prevalence of dysphagia showed a wide range from 3% to 63% [16,17,18,19]. Since the onset of dysphagia is highly related to motility and structural disorders, such as stroke or Parkinson’s disease [11], most studies addressing dysphagia have focused on individuals admitted to nursing homes or hospitals [9,20]. However, in recent years, the prevalence of dysphagia has been fairly high, even in older adults without any causative diseases or disorders [3]. A study by González-Fernández et al. [21], which evaluated the prevalence of dysphagia using the water-swallowing test, confirmed that dysphagia was present in many elderly female patients who did not have a neurological disease or a history of dysphagia. Other studies reported that some older adults considered swallowing problems a symptom of the normal aging process and did not seek proper diagnosis or treatment; therefore, a significant number of patients who experienced dysphagia symptoms did not report these symptoms to their doctors [22,23,24]. Achem and Devault [25] discovered physiological changes related to aging in areas involved in the swallowing function, not only in older adults with signs of dysphagia but also in older adults without these signs. In addition, the number of older adults is increasing significantly worldwide; in particular, South Korea is experiencing a more rapid rate of population aging than other countries [26]. Considering these circumstances, further studies should be conducted to discover additional risk factors for dysphagia that are easily overlooked in older, healthy, community-dwelling adults. In addition, because the oral cavity plays an important role in the chewing and swallowing processes [14,27], a more comprehensive evaluation of oral health conditions is needed to further elucidate potential risk factors in the oral cavity. 

In this context, we aimed to evaluate the risk of dysphagia and identify the association between dysphagia and oral health conditions, as well as the strength of the association, in older, healthy, community-dwelling adults aged 65 years or older without any history related to causative diseases of dysphagia.

## 2. Materials and Methods

### 2.1. Participants

**Figure 1:**
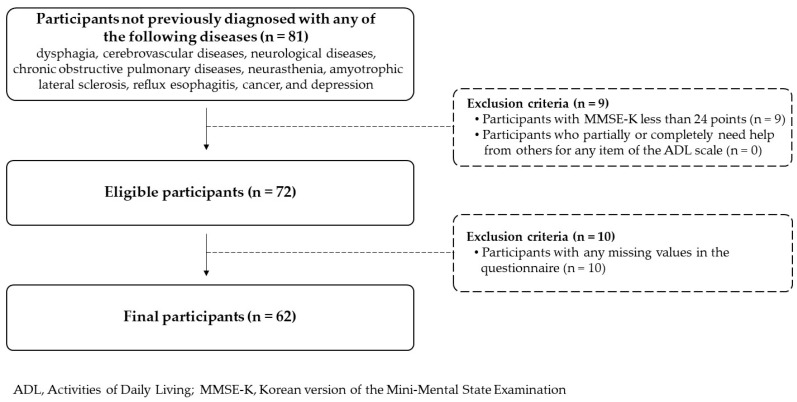
Flowchart of participant selection.

This study was approved by the Institutional Review Board of Gachon University (approval no. 1044396-201904-HR-065-01). This study was conducted in accordance with the principles of the Declaration of Helsinki. To recruit participants, 10 senior welfare facilities located in Yeonsu-gu, Incheon, South Korea, were selected by convenience sampling. The purpose and methods of this study were fully explained to adults aged 65 years or older who visited the selected facilities from 1 July 2019 to 30 January 2020. Those who were diagnosed with the following diseases or disorders were excluded from the study: dysphagia, cerebrovascular diseases such as stroke, neurological diseases such as Parkinson’s disease, chronic obstructive pulmonary diseases such as chronic bronchitis, neurasthenia, amyotrophic lateral sclerosis, reflux esophagitis, cancer, and depression. Informed consent was obtained from 81 individuals who understood the research process and voluntarily agreed to participate in this study. The Korean version of the Mini-Mental State Examination was used to screen the cognitive status of all volunteers [28]. Data from participants (n = 9) who scored lower than 24 points, the cutoff value for detecting cognitive impairment, were excluded. After exclusion, independence in seven activities of daily living, such as dressing and eating, was evaluated using the activities of daily living scale [29]. Since all 72 participants responded that they could perform all seven daily activities without the help of others, we judged that all participants were able to perform basic activities of daily living independently. After all the data were collected, the data from participants (n = 10) who had missing values for at least one question were excluded. Data from 62 participants (51 women, 11 men) were finally analyzed (Figure 1). The mean age of the participants was 75.61 ± 7.79 years. 

### 2.2. Measurements 

A trained examiner administered the questionnaire to all the participants. Subsequently, oral examinations and evaluations of various oral health conditions, including orofacial muscle strength, were performed. We collected four types of data: sociodemographic factors (age, sex, education level, employment status, and living status), general health-related characteristics (systemic disease status, alcohol consumption, and smoking status), oral health conditions (perceived oral health condition, subjective oral discomfort (masticatory discomfort and pronunciation discomfort due to problems in the mouth, history of toothache within the last month), need for prosthetic treatment, denture-related conditions (wearing dentures or not, denture type, denture discomfort), unstimulated salivary flow rate, the number of remaining teeth excluding the third molar and exposed tooth roots, the number of functional tooth units, orofacial muscle strength (anterior tongue elevation, buccinator muscle, and lip strength), and the risk of dysphagia. 

The number of functional tooth units was defined as the number of pairs of natural or artificial posterior teeth that opposed each other during chewing [30]. The Iowa Oral Performance Instrument (IOPI) (IOPI Medical, Redmond, WA, USA) was used to measure orofacial muscle strength [31,32]. The strength of all the orofacial muscles was measured according to the IOPI guidelines. The participants were instructed to sit on a chair, straighten their backs, and lean lightly on the backrest with their feet pelvic width apart and their eyes gazing forward. The strength of each muscle was measured three times consecutively, and the highest value was recorded. To measure the anterior tongue elevation strength, the bulb was positioned longitudinally on the hard palate immediately behind the alveolar ridge. Participants were instructed to close their mouth slightly and press the bulb with the front part of their tongue with maximum effort for 2 s. 

According to the criteria provided by the IOPI manufacturer [33], those with scores lower than 34 kPa were classified into the weak anterior tongue elevation strength group, and those with scores of 34 kPa or higher were classified into the normal group. To measure the strength of the buccinator muscle, the bulb was positioned laterally between the cheek on the side, usually used for mastication, and the buccal surface of the teeth. The participants were instructed to lightly occlude their teeth and squeeze their cheek muscles against the bulb with maximum effort. Those with scores lower than 19 kPa, which was the mean value of the participants, were classified into the weak buccinator muscle strength group, whereas those who scored 19 kPa or higher were classified into the normal group. Finally, to measure lip strength, two wooden tongue blades were used to prevent the bulb from being bitten by the teeth [31]. The bulb was inserted between the two tongue blades and positioned between the lips at the dental midline. When the bulb sandwiched between the two tongue blades was placed between the lips, the participants were instructed to close their lips lightly and protrude them slightly. They were then instructed to squeeze the bulb with their lips with maximum effort for 2 s. Using their average as the threshold, those with scores lower than 12 kPa were classified into the weak lip strength group, and those who scored 12 kPa or higher were classified into the normal group. 

For hyposalivation diagnosis, the unstimulated whole salivary flow rate was measured in the participants [34]. The participants were instructed in advance not to eat or perform any oral hygiene activities, such as tooth brushing, at least 2 h before saliva collection. The saliva was collected for 5 min using the spitting method. Those who recorded less than 0.1 mL/min were classified into the hyposalivation group, and those who had 0.1 mL/min or more were classified into the normal group [35]. 

Finally, the Dysphagia Risk Assessment Scale (DRAS) was used to screen for dysphagia risk [36,37]. The DRAS is a tool developed for use with older community-dwelling adults, and its validity has been demonstrated in the literature [19,38]. The DRAS consists of 23 questions in four sections: oral preparatory and oral dysphagia (eight questions including “food remains in your cheek after swallowing”), pharyngeal dysphagia (seven questions including “food or liquid goes into your nasal cavity while eating”), esophageal dysphagia (three questions including “food or sour liquid comes back up into your throat from the stomach”), and aspiration (five questions including “choking or coughing during swallowing”). Each question was scored from 0 (never) to 3 (always). The total score ranged from 0 to 69. Those with a score lower than 6 points were classified into the normal group, and those with a score of 6 or higher were classified into the high-risk group for dysphagia [37,39].

### 2.3. Statistical Analysis 

The collected data were analyzed using SPSS (ver. 23; IBM Corp., Armonk, NY, USA), and the significance level was set at *p* < 0.05. To analyze the differences in dysphagia risk according to sociodemographic factors, general health-related characteristics, and oral health conditions, the Mann–Whitney U test or Kruskal–Wallis test was conducted. After controlling for potential confounders associated with dysphagia risk, a multiple logistic regression analysis was used to analyze the strength of the association between oral health conditions and dysphagia risk. In the final logistic regression model, the dependent variable was dysphagia risk, and the factors that were *p* < 0.05 in the bivariate analysis were used as independent variables.

## 3. Results

### 3.1. Dysphagia Risk among Participants

Based on the DRAS, 38.7% (n = 24) of participants belonged to the high-risk group for dysphagia. The mean DRAS score was 9.21 ± 2.68 and 2.53 ± 1.68 points in the high-risk dysphagia and normal groups, respectively (*p* < 0.001). The high-risk group showed a significantly higher risk than that of the normal group in all sections of the DRAS (*p* < 0.001) except for esophageal dysphagia (*p* = 0.178) (Table 1).

### 3.2. Dysphagia Risk according to Sociodemographic Characteristics and General Health-Related Characteristics

The risk of dysphagia was higher in patients who were aged 75 years or older (6.62 ± 3.34), whose highest education level was primary school or lower (6.25 ± 4.05), and who were not economically active (5.95 ± 3.87) than that of each respective control group (*p* < 0.05). The risk level tended to be lower in those who lived with their spouse than in those who lived alone or with their children; however, the difference was not significant (*p* = 0.065). In addition, although no significant difference was observed between dysphagia risk and all the general health-related characteristics, the risk level of dysphagia tended to be higher in those who had two or more systemic diseases (5.57 ± 3.73) (*p* = 0.078) (Table 2).

### 3.3. Dysphagia Risk According to Oral Health Conditions

The risk of dysphagia was higher in those who reported “difficulty chewing due to problems in the mouth” (8.58 ± 3.77) or “difficulty pronouncing” (10.56 ± 2.40) than that of each control group (*p* < 0.01). The risk level was higher in groups who had hyposalivation (6.71 ± 3.94), who had less than 10 remaining teeth (7.38 ± 4.15), and who had 0–3 functional tooth units (10.20 ± 3.70) than that of each respective control group (*p* < 0.05). Furthermore, the groups with weak anterior tongue elevation strength and weak buccinator muscle strength (6.55 ± 3.73 and 6.64 ± 3.60 kPa, respectively) showed a higher risk of dysphagia than the respective normal groups (*p* = 0.020 and 0.002, respectively) (Table 3).

### 3.4. Influence of Oral Health Conditions on Dysphagia Risk 

In the final logistic regression model adjusted for sociodemographic characteristics, masticatory discomfort, hyposalivation, and weak buccinator muscle strength were identified as factors that increased the risk of dysphagia (*p* < 0.05) (Table 4). In addition, the probability of belonging to the high-risk group for dysphagia increased as participants felt more uncomfortable pronouncing words because of oral problems (*p* = 0.059). We confirmed that there was no multicollinearity between the independent variables in the final adjusted model (variance inflation factor < 10, tolerance > 0.10). The model’s Nagelkerke R^2^ was 0.654, and the Hosmer and Lameshow test results revealed a good fit.

## 4. Discussion

Dysphagia, or swallowing impairment, is a health risk that threatens human dignity because swallowing is essential for sustaining an individual’s life [1]. Considering the fairly high prevalence of dysphagia in older adults without any dysphagia-inducing disease in recent years [3], attempts should be made to identify potential risk factors for swallowing impairment in older community-dwelling adults. Although a small number of studies have suggested that oral problems such as tooth loss, which becomes more frequent with age, have a significant impact on the deterioration of swallowing function [12,27], the effects of oral health conditions, a factor that can be treated, are receiving less attention than other factors. Therefore, this study examined healthy adults aged 65 years or older who were independently living in the community to evaluate their dysphagia risk and analyzed the oral health conditions associated with dysphagia risk and their influence. 

First, the results showed that 39% of the participants belonged to the high-risk group for dysphagia. A recent systematic review and meta-analysis [40] estimated the overall prevalence of dysphagia to be approximately 33% in older adults, with a wide range from 12 to 34% in older community-dwelling adults, depending on the tool used. A study that used the same tool as that used in the present study [41] reported that approximately 53% of participants were at risk of dysphagia. In another study, the prevalence of dysphagia was about 18% and 22% in men and women, respectively [42]. Although our study selected only older adults who could perform basic activities of daily living independently without a history of cerebrovascular diseases and scored within the normal range on the dementia screening test, a significant number of the participants were at risk of dysphagia. While there are various causes of dysphagia in older community-dwelling adults, aging itself causes degeneration or reduced flexibility of the muscles or structures involved in swallowing, such as the pharynx, which eventually leads to reduced swallowing efficiency [7,43]. Physiological changes related to aging have been reported, especially in the upper esophageal sphincter and pharyngeal region, and esophageal sensation becomes markedly dull with age [25,43]. In addition, sarcopenia, a condition of loss of muscle mass and quality with age, can directly affect the strength of the muscles used for swallowing and cause dysphagia, especially oropharyngeal function decline [44]. Globally, population aging is accelerating. According to Statistics Korea, South Korea is the fastest-aging country in the world and is expected to become a super-aged society by 2025, where adults aged 65 years and older account for more than 20% of the total population [45]. A study using the Korean National Health Insurance Service database reported that the prevalence of dysphagia that requires medical attention showed a noticeable increase every year, especially in older adults, and that the mortality rate of the dysphagia group was approximately three times higher than that of the non-dysphagia group [46]. Therefore, we suggest that dysphagia, a disorder prevalent among older, healthy, community-dwelling adults, is an important issue that must be considered in an aging society. In addition, to minimize the physical and psychological complications caused by dysphagia, there is a need to screen older community-dwelling adults for the risk of dysphagia using validated screening tools to detect those at risk of dysphagia early and provide them with appropriate interventions. 

Second, in the final multivariate logistic regression model adjusted for sociodemographic characteristics, this study found that individuals with subjective masticatory discomfort, hyposalivation, and weak buccinator muscle strength had a significantly higher risk of dysphagia (*p* < 0.05). In particular, buccinator muscle weakness had the strongest association with the risk of dysphagia (odds ratio = 11.108). The buccinator muscle is the main square-shaped facial muscle underneath the cheek that participates in chewing and normal swallowing [2]. Various muscles and cranial nerves are involved in the chewing and swallowing processes; in particular, orofacial muscles such as the orbicularis oris and buccinator muscles play diverse roles in the oral phase of swallowing [32]. For the oral phase to occur successfully, the lips must be closed, tongue movement must be smooth, and the various orofacial muscles involved in the chewing process must function normally [2,31]. In addition, the buccinator muscle helps mastication to occur efficiently by lifting the bolus to the occlusal surface of the teeth and pulling back the corners of the mouth to close the lips [47,48,49]. Buccinator muscle strength is reportedly a sensitive measure that can reflect the degree of orofacial weakness, and prolonged cheek compression lowers the chance of swallowing problems [50]. Therefore, factors that can weaken the buccinator muscle in older adults, such as tooth loss, should be monitored regularly, and effective intervention programs that can help maintain muscle strength should be provided considering individual characteristics. Park et al. [51] reported that expiratory muscle strength training significantly improved the strength of the buccinator muscle in older community-dwelling adults. In addition, our study reaffirmed that hyposalivation increased the risk of dysphagia, as demonstrated in a previous study [52]. For the objective diagnosis of oral dryness in our study, the unstimulated salivary flow rate was measured in the participants [53]. While the normal unstimulated salivary flow rate is approximately 0.3–0.4 mL/min [54], the mean salivary flow rate of the participants was fairly low (0.11 ± 0.72 mL/min), and approximately 45% of the participants were found to have hyposalivation. Generally, approximately 1.5 L of saliva is secreted per day by normal individuals and is not secreted without stimulation [2]. Saliva secretion gradually decreases with increasing age and the number of drugs taken [55]. Saliva plays an important role in maintaining oral health and ecological balance and, more importantly, contributes greatly to the mastication and swallowing processes [3,54]. Saliva enhances taste and initiates the digestive process [54]. To form a soft food bolus suitable for swallowing, saliva must be sufficiently secreted during mastication, and the food must be mixed well with the secreted saliva [56]. Moreover, a pliable oral mucosa sufficiently covered with salivary secretions is required for the food bolus to move smoothly toward the pharynx [57]. Furthermore, a cross-sectional study demonstrated that oral dryness contributed to dysphagia more than aging or comorbidity in community-dwelling adults aged 50 years and older [42]. Another study reported that patients with dysphagia complained of a lower unstimulated salivary flow rate and took more than twice as long in the oral phase of swallowing as the normal group [58]. Additionally, hyposalivation accelerates the development of dental caries and periodontal disease, which are the main causes of tooth loss and risk factors for dysphagia [59]. Thus, for both oral health and dysphagia prevention, symptoms of oral dryness should be regularly monitored, and various interventions that can promote salivary secretion should be provided at an early stage. Raj et al. [60] reported that oral exercise can increase salivary secretion by moving various muscles, including the buccinator and orbicularis oris. Finally, our study found that older adults with weak anterior tongue elevation or fewer than 10 remaining teeth had a higher risk of dysphagia than the respective control groups in the bivariate analysis (*p* = 0.020 and 0.038, respectively) but not in our final multivariate logistic regression model (*p* > 0.05). However, several studies targeting nursing home residents have identified weak tongue pressure as a clinical indicator that is predictive of dysphagia development [61,62]. The tongue is a complex muscular organ that plays an important role in manipulating food and propelling it from the oral cavity into the pharynx during swallowing [63]. Generally, the force-generating capacity of the oral tongue decreases with age, and this change can lead to reduced pressure and poor bolus clearance during the oral phase [64,65]. In addition, a cross-sectional observational study reported that the loss of posterior tooth occlusion increased the risk of dysphagia in older nursing home residents [12]. Another study suggested that the type of prosthesis and number of teeth were associated with oropharyngeal dysphagia [13]. Unlike previous studies that examined older adults with a causative disease of dysphagia, we surmised that tongue elevation strength and the number of remaining teeth may be similar in the participants and that the two factors had no impact on the decline in swallowing function in the adjusted final regression model. However, further research is needed to confirm the association between tongue strength, number of remaining teeth, and dysphagia risk in older healthy adults. 

Most previous studies on the prevalence of dysphagia or its risk factors examined individuals with cerebrovascular diseases such as stroke or neurological diseases such as Parkinson’s disease. The significance of the present study lies in the fact that subjective masticatory discomfort, hyposalivation, and weak buccinator muscles were associated with dysphagia in older, healthy, community-dwelling adults who could independently perform basic activities of daily living without any causative diseases of dysphagia. In particular, since the oral cavity plays a very important role as the first organ in the swallowing process, and oral health can be intervened, this study tried to evaluate oral health conditions more comprehensively. The findings of this study will contribute to the establishment of effective intervention strategies that can enable early detection of older, healthy, community-dwelling adults at a high risk of dysphagia and help manage their ability to eat independently. 

Despite these strengths, this study has several limitations. First, the generalizability of our findings may be limited because the final sample size was small after excluding those with a history of dysphagia-inducing disease and because this study only examined older adults in a certain region. Second, the gender distribution was not even among the participants. Because orofacial muscle strength can vary depending on demographic factors, including gender [31,66], the final regression model was controlled for sociodemographic characteristics. Regardless of the effort, the findings of this study may differ from those of previous studies due to the uneven sex distribution. Third, because this was a cross-sectional study, proving a causal relationship between the variables was difficult, especially between dysphagia and oral health conditions. Fourth, because no standard was provided by the IOPI manufacturer, the mean value of the participants was used as the cutoff value for orofacial muscle weakness, except for the tongue strength. Generally, the normal range of muscle strength varies depending on various factors, including ethnicity [67]. Thus, the association with dysphagia may differ depending on the cutoff value used for orofacial muscle strength. This highlights the need for further research to establish reference values for normal orofacial muscle strength in healthy older Korean adults. Finally, notwithstanding that this study excluded older adults with dysphagia-inducing disease, we could not reflect several general health conditions, including gastrointestinal disorders and esophageal inflammation, that may cause dysphagia. Hence, a longitudinal study with a larger sample size is required to elucidate the potential risk factors for dysphagia in older, healthy, community-dwelling adults. 

## 5. Conclusions

This cross-sectional study found that approximately 40% of older, healthy, independent, community-dwelling adults without a history of any causative disease for dysphagia, including cerebrovascular disease, belonged to a high-risk group for dysphagia. The oral health-related factors independently associated with dysphagia were weak buccinator muscle strength, hyposalivation, and subjective masticatory discomfort. Based on the findings of our study, we suggest that the swallowing function of older community-dwelling adults should be periodically screened. These findings suggest that intervention programs that promote salivary secretion and enhance buccinator muscle strength can significantly contribute to the recovery or maintenance of swallowing function.

## Figures and Tables

**Table 1:** Dysphagia risk in each section of DRAS.

DRAS Category	Dysphagia Risk		*p*-Value
	Normal Group (n = 38)	High-Risk Group (n = 24)	
Oral preparatory and oral dysphagia	1.37 ± 1.38	5.83 ± 2.09	<0.001
Pharyngeal dysphagia	0.05 ± 0.32	0.71 ± 0.95	<0.001
Esophageal dysphagia	0.47 ± 0.72	0.79 ± 0.93	0.178
Aspiration	0.63 ± 0.88	1.88 ± 1.36	<0.001
Total score	2.53 ± 1.68	9.21 ± 2.68	<0.001

Analyzed by Mann–Whitney test. All values are expressed as means ± standard deviations. The high-risk dysphagia group included participants who scored 6 points or higher on the DRAS. DRAS, Dysphagia Risk Assessment Scale.

**Table 2:** Dysphagia risk according to sociodemographic characteristics and general health-related characteristics.

Variables		n	Dysphagia Risk	z or x^2^	*p*-Value
Sex	female	51	5.49 ± 4.06	−1.573	0.116
	male	11	3.36 ± 2.46		
Age, years ^†^	<75	33	3.79 ± 3.91	−3.386	< 0.001
	≥75	29	6.62 ± 3.34		
Education level	≤primary school	32	6.25 ± 4.05	−2.369	0.018
	≥middle school	30	3.90 ± 3.38		
Employment status	worker	21	3.48 ± 3.48	−2.666	0.008
	non-worker	41	5.95 ± 3.87		
Living status	alone	8	6.00 ± 4.72	5.476	0.065
	with spouse	26	3.69 ± 3.00		
	with children	28	6.18 ± 4.11		
Smoking status	non-smoker	54	5.44 ± 4.02	−1.708	0.088
	smoker (former and current)	8	2.88 ± 1.88		
Alcohol consumption	non-drinker	48	5.38 ± 4.01	−1.040	0.298
	drinker	14	4.21 ± 3.46		
No. of systemic disease	0–1	18	4.00 ± 4.17	−1.760	0.078
	≥2	44	5.57 ± 3.73		
Hypertension	yes	42	5.67 ± 4.04	−1.543	0.123
	no	20	3.95 ± 3.37		
Hyperlipidemia	yes	30	5.27 ± 4.25	−0.028	0.977
	no	32	4.97 ± 3.59		
Diabetes	yes	16	5.19 ± 3.01	−0.493	0.622
	no	46	5.09 ± 4.19		
Osteoporosis	yes	14	5.00 ± 4.35	−0.296	0.767
	no	48	5.15 ± 3.80		
Cardiovascular disease	yes	13	6.15 ± 3.18	−1.502	0.133
	no	49	4.84 ± 4.05		

Analyzed by Mann–Whitney test or Kruskal–Wallis test. All values are expressed as means ± standard deviations. ^†^ Divided by participants’ average. Only systemic diseases with a prevalence of 20% or higher are presented. No., number.

**Table 3:** Dysphagia risk according to oral health conditions.

Variables		n	Dysphagia Risk	z or x^2^	*p*-Value
Perceived oral health status	good	22	4.59 ± 3.26	5.538	0.063
	fair	26	4.50 ± 4.29		
	poor	14	7.07 ± 3.66		
Masticatory discomfort	no (comfortable)	39	3.72 ± 3.24 ^a^	16.267	<0.001
	fair	8	5.50 ± 3.29 ^ab^		
	uncomfortable	15	8.58 ± 3.77 ^b^		
Pronunciation discomfort	no (comfortable)	50	4.28 ± 3.45 ^a^	12.567	0.002
	fair	7	7.14 ± 4.18 ^ab^		
	uncomfortable	5	10.56 ± 2.40 ^b^		
History of toothache	no	43	4.33 ± 3.24	4.317	0.115
within the last month	yes (moderate)	7	6.29 ± 3.98		
	yes (severe)	12	7.25 ± 5.20		
Need for prosthetic	yes	24	4.76 ± 3.88	−1.067	0.286
treatment	no	38	5.67 ± 3.94		
Type of dentures	upper or lower dentures	8	5.63 ± 5.31	−1.530	0.140
	upper and lower dentures	13	8.15 ± 3.73		
Frequency of denture	0	4	4.75 ± 5.12	2.253	0.324
discomfort ^†^	1	11	6.91 ± 3.61		
	≥2	6	9.33 ± 5.20		
Unstimulated salivary	hyposalivation (<0.1 mL/min)	28	6.71 ± 3.94	−3.054	0.002
flow rate	normal	34	3.79 ± 3.38		
No. of remaining teeth	<10	13	7.38 ± 4.15 ^a^	6.541	0.038
	10–19	12	5.83 ± 4.46 ^ab^		
	≥20	37	4.08 ± 3.28 ^b^		
No. of functional tooth	0–3	5	10.20 ± 3.70 ^a^	7.718	0.021
units	4–5	18	4.72 ± 2.98 ^b^		
	6	39	4.64 ± 3.91 ^b^		
Anterior tongue elevation	weak (<34 kPa)	22	6.55 ± 3.73	−2.334	0.020
strength ^†^	normal (≥34 kPa)	40	4.33 ± 3.80		
Buccinator muscle	weak (<19 kPa)	28	6.64 ± 3.60	−3.111	0.002
strength ^††^	normal (≥19 kPa)	34	3.85 ± 3.72		
Lip strength ^††^	weak (<12 kPa)	26	5.96 ± 3.87	−1.597	0.110
	normal (≥12 kPa)	36	4.50 ± 3.85		

Analyzed by Mann–Whitney test or Kruskal–Wallis test. ^a^,^b^ The same characters are not significant by Bonferroni’s multiple comparisons at α = 0.05. All values are expressed as mean ± standard deviations. ^†^ Classified based on IOPI criteria. ^††^ Classified using the mean value of participants as cutoff value. IOPI, Iowa Oral Performance Instrument; No., number.

**Table 4:** Oral health conditions associated with dysphagia risk based on multiple logistic regression analysis.

Characteristics	OR (CI)	*p*-Value *
Masticatory discomfort (ref. very comfortable)	2.292 (1.013–5.184)	0.046
Pronunciation discomfort (ref. very comfortable)	2.790 (0.961–8.099)	0.059
Hyposalivation (ref. normal)	9.255 (1.142–74.991)	0.037
≥20 remaining teeth (ref. < 20)	0.910 (0.140–5.900)	0.921
6 functional teeth units (ref. ≤ 5)	0.590 (0.107–3.258)	0.546
Weak buccinator muscle strength (ref. normal)	11.108 (1.224–100.845)	0.032
Weak tongue elevation strength (ref. normal)	0.066 (0.004–1.207)	0.067

Factors with *p* < 0.05 in the bivariate analysis were used in the model as independent variables. * Adjusted for sociodemographic characteristics (age, sex, employment status, and education level). Division of characteristics: masticatory discomfort—very comfortable (1), comfortable (2), more or less (3), uncomfortable (4), and very uncomfortable (5); pronunciation discomfort—very comfortable (1), comfortable (2), more or less (3), uncomfortable (4), and very uncomfortable (5); unstimulated salivary flow rate—normal (1), hyposalivation (2); no. of remaining teeth—≤19 (1), ≥20 (2); no. of functional teeth units—≤5 (1), 6 (2); buccinator muscle strength—normal (1), weak (2); tongue elevation strength—normal (1), weak (2). OR, odds ratio; CI, 95% confidence interval.

## Data Availability

The data presented in this study are available on request from the corresponding author.
